# Preschool Weight and Body Mass Index in Relation to Central Obesity and Metabolic Syndrome in Adulthood

**DOI:** 10.1371/journal.pone.0089986

**Published:** 2014-03-03

**Authors:** Lise Graversen, Thorkild I. A. Sørensen, Liselotte Petersen, Ulla Sovio, Marika Kaakinen, Annelli Sandbaek, Jaana Laitinen, Anja Taanila, Anneli Pouta, Marjo-Riitta Järvelin, Carsten Obel

**Affiliations:** 1 Section for General Medical Practice, Department of Public Health, Aarhus University, Aarhus, Denmark; 2 Institute of Preventive Medicine, Bispebjerg and Frederiksberg University Hospital, The Capital Region, Copenhagen, Denmark; 3 Novo Nordisk Foundation Center for Basic Metabolic Research, Faculty of Health and Medical Sciences, University of Copenhagen, Copenhagen, Denmark; 4 National Centre for Register-Based Research, Faculty of Social Sciences, Aarhus University, Aarhus, Denmark; 5 Department of Obstetrics and Gynaecology, University of Cambridge, Cambridge, United Kingdom; 6 Department of Epidemiology and Biostatistics, Imperial College, London, United Kingdom; 7 Institute of Health Sciences, University of Oulu, Oulu, Finland; 8 Biocenter Oulu, University of Oulu, Oulu, Finland; 9 Finnish Institute of Occupational Health, Oulu, Finland; 10 Primary Health Care Unit, University Hospital of Oulu, Oulu, Finland; 11 National Institute of Health and Welfare, Oulu, Finland; 12 Department of Obstetrics and Gynecology, University of Oulu and Oulu University Hospital, Oulu, Finland; University College London, United Kingdom

## Abstract

**Background:**

If preschool measures of body size routinely collected at preventive health examinations are associated with adult central obesity and metabolic syndrome, a focused use of these data for the identification of high risk children is possible. The aim of this study was to test the associations between preschool weight and body mass index (BMI) and adult BMI, central obesity and metabolic alterations.

**Methods:**

The Northern Finland Birth Cohort 1966 (NFBC1966) (N = 4111) is a population-based cohort. Preschool weight (age 5 months and 1 year) and BMI (age 2–5 years) were studied in relation to metabolic syndrome as well as BMI, waist circumference, lipoproteins, blood pressure, and fasting glucose at the age of 31 years. Linear regression models and generalized linear regression models with log link were used.

**Results:**

Throughout preschool ages, weight and BMI were significantly linearly associated with adult BMI and waist circumference. Preschool BMI was inversely associated with high-density lipoprotein levels from the age of 3 years. Compared with children in the lower half of the BMI range, the group of children with the 5% highest BMI at the age of 5 years had a relative risk of adult obesity of 6.2(95% CI:4.2–9.3), of adult central obesity of 2.4(95% CI:2.0–2.9), and of early onset adult metabolic syndrome of 2.5(95% CI:1.7–3.8).

**Conclusions:**

High preschool BMI is consistently associated with adult obesity, central obesity and early onset metabolic syndrome. Routinely collected measures of body size in preschool ages can help to identify children in need of focused prevention due to their increased risk of adverse metabolic alterations in adulthood.

## Introduction

Childhood overweight and obesity have serious public health implications, as they are linked to adverse health outcomes in childhood, and they track into adulthood [1]–[5]. Both general and central obesity in adulthood are associated with increased mortality [6]–[9]. Several studies have linked childhood BMI to adverse metabolic alterations and metabolic syndrome in adulthood [1], [10]–[12]. However, so far the studies have focused on school-age children and complex measures of childhood growth such as BMI peak, BMI rebound, growth peak velocity and z-score changes [13]–[15].

In many countries, preschool children attend routine health examinations, where height and weight are measured frequently by pediatricians or general practitioners. For a targeted use of these measurements, we need detailed knowledge about long-term health consequences of excess weight in early childhood. Previously, we have shown that preschool weight and BMI are closely associated with adolescent overweight, and that the risk linked to these risk indicators is stable over the developing obesity epidemic (submitted to PLoSONE). In addition to the health implications of their link to adolescent overweight, the preschool weight and BMI measures may be associated with even more severe health implications like metabolic alterations in adulthood. Metabolic alterations in adulthood are known to be linked to disease development and mortality [16]. The identification of children at risk of these alterations as early as in preschool ages, followed by well-designed preventive interventions, could provide a considerable public health potential. The Northern Finland Birth Cohorts offer a unique opportunity of studying the relation between preschool growth and adult outcomes owing to extensive growth measurements in early childhood and data from a comprehensive adult clinical examination on a large number of individuals.

The aim of this study was to test the associations between preschool weight (age 5 months and 1 year) and BMI (each year from age 2–5) and adult BMI, central obesity and metabolic alterations at the age of 31 years. We tested preschool body measures continuously to maximize data utilization and categorized it to maximize the clinical value, and identify a group of children at high risk.

## Material and Methods

### Study population

The Northern Finland Birth Cohort 1966 (NFBC 1966) consists of 96.3% of all children who were due to be born in the provinces of Oulu and Lapland in Northern Finland in 1966, and

**Figure 1:**
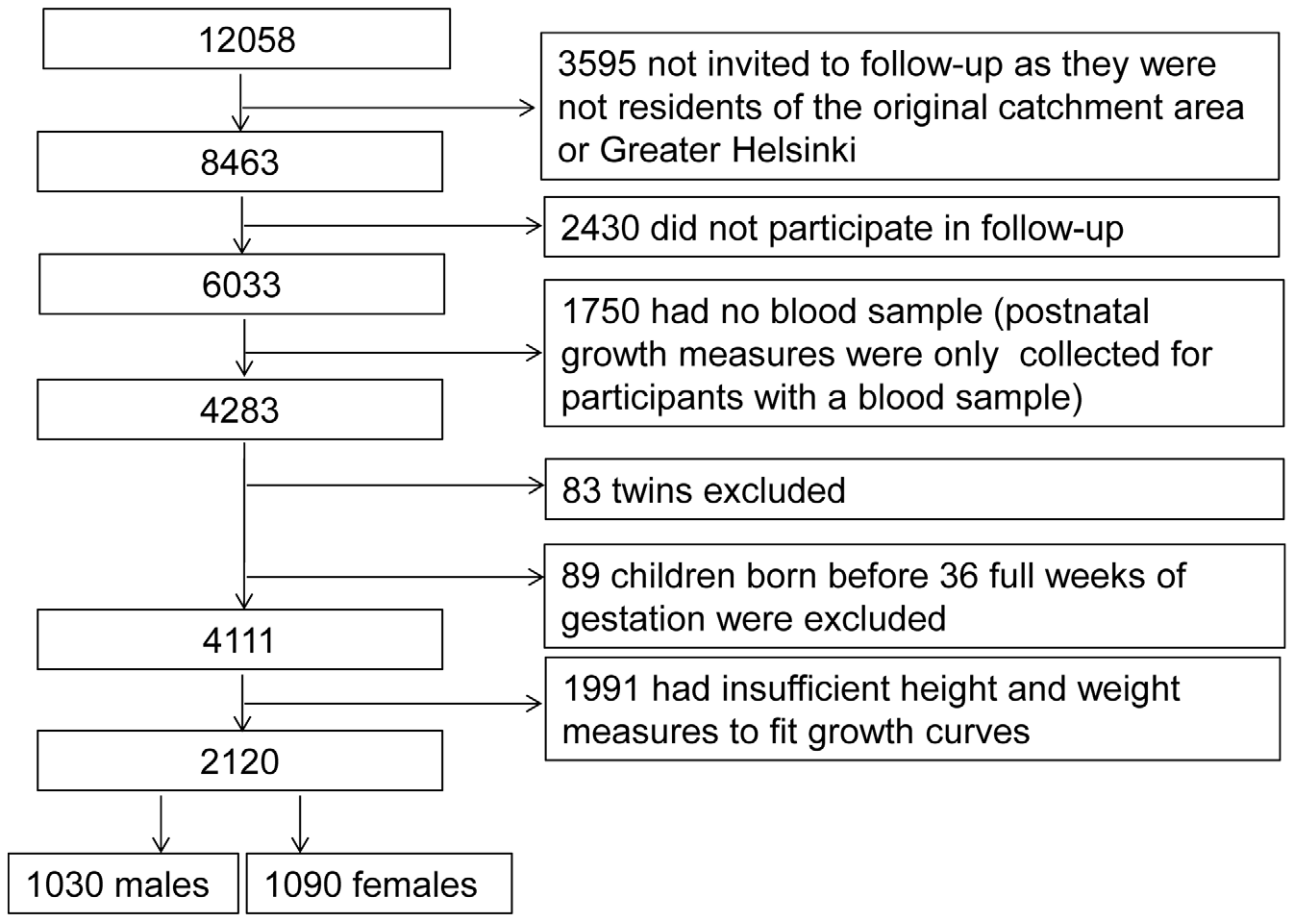
Flow chart of the study population.

12058 live-born children entered the study [17], [18]. Data collection was started in pregnancy via a structured self-completed questionnaire concerning health and the family's social situation. Birth data were coded from hospital records at the time of delivery. When the subjects living in the original target area or in the capital area (N = 8463) were 31 years of age, they were invited to participate in a follow-up study including a clinical examination, and 71% of the invited took part (N = 6033). The clinical examination included measurement of weight and height, waist circumference (midway between the lowest rib margin and the iliac crest), and systolic and diastolic blood pressure (taken by trained nurses using a standard mercury sphygmomanometer after 15 minutes of rest) [18] and drawing of a blood sample after overnight fasting. Samples were stored at −70°C until analyzed. Enzymatic assays of fasting serum glucose, high density lipoprotein (HDL), and triglyceride concentrations were carried out using a Hitachi 911 automatic analyzer and commercial reagents (Boehringer Mannheim, Mannheim, Germany) in the accredited laboratory of Oulu University Hospital (Oulu, Finland). DNA was analyzed for 5753 subjects. For those with a DNA sample available, data on their postnatal growth were obtained from scans of the original health clinic records (N = 4283). For the analyses of the present study, we excluded children born before 36 full weeks of gestation were completed. Antenatal data and postnatal growth data were available for 4111 singletons in the NFBC1966 cohort (Figure 1). Almost full data on metabolic risk factors were available. Signed, informed consent and written permission was obtained from the study participants at the age of 31. The University of Oulu Ethics Committee approved the study.

### Growth measures

On average, 20 height- and weight measurements per child were available from birth until adolescence. Individual growth curves were fitted from infancy till the age of 5.5 years (File S1). Weight and height at specific time points were extracted from these fitted growth curves. We used measurements after birth (birth length and weight not included) until the age of 5.5 years to estimate curves for all children with at least one measurement before the age of 6 months, one measurement between 6 months and 1.5 years, one measurement between 1.5 and 4 years, and another between 4 and 5.5 years. Sufficient data to estimate growth curves were available for 2120 children. At the age of 5 months and 1 year, weight was extracted, and at the age of 2, 3, 4 and 5 years height and weight were extracted to calculate BMI, as these measures are generally used in clinical practice at these ages.

### Metabolic syndrome

We used the International Diabetes Federation's definition of metabolic syndrome [19]. This definition is based on high waist circumference (>94 cm in males and ≥80 cm in females) and two of the following four risk factors; 1) reduced HDL (<1.04 mmol/l in males and <1.29 mmol/l in females), 2) increased triglyceride (≥1.7 mmol/l), 3) increased fasting glucose (≥5.6 mmol/l or being on diabetes medication), and 4) increased blood pressure (SBP ≥130 mmHg or DBP ≥85 mmHg or being on antihypertensive medication).

### Statistical analyses

**Figure 2:**
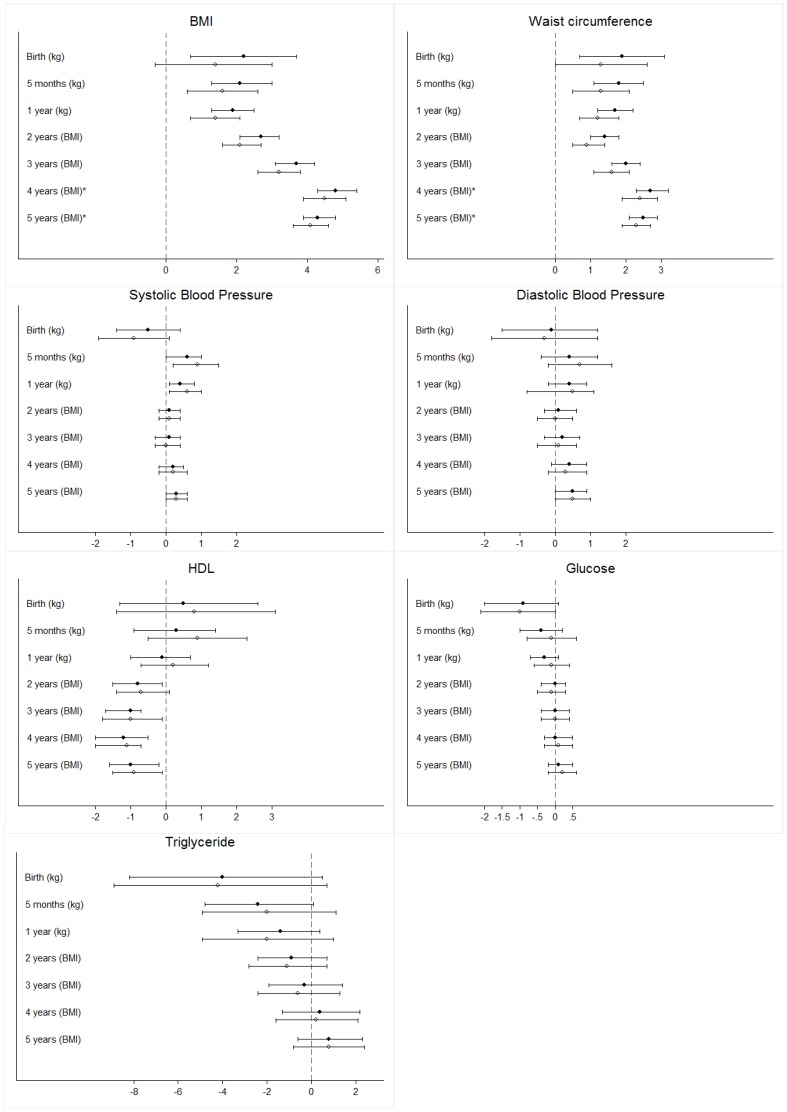
Relationship between preschool body size and body mass index (BMI), waist circumference, blood pressure, HDL, glucose and triglyceride at the age of 31 years. Footnote: Differences in the dependent variable in adulthood per kg or BMI unit in childhood are shown as percentages (x axis). We used the formula (10^(β)^−1) ×100 to present percentage differences. Unadjusted associations (solid dots) and associations adjusted for birth weight, gestational week, maternal smoking during pregnancy, maternal age at birth, maternal pre-pregnancy BMI, maternal education and parity (circles) are presented with their 95% confidence interval. * represents a statistically significant difference between genders.

Basic characteristics of the variables included are reported as medians (with their 5–95% range) or percentages (Table 1). We tested the linear associations between weight at birth, at 5 months and 1 year and BMI each year from 2 to 5 years and adult BMI, waist circumference, HDL, triglyceride, fasting glucose, diastolic and systolic blood pressure. We used a linear regression model, and outcomes were log-transformed to reduce skewness. Linear correlations, linear correlations with a quadratic component and splines were tested. Quadratic components and splines failed to improve the fit in any of the models and therefore only linear models are shown. The models were evaluated using R^2^. We studied males and females separately, but pooled the data when there was no statistically significant indication of interaction. Where genders were analyzed together, the estimates were adjusted for gender. The linear associations were adjusted for birth weight (kg), gestational age (weeks), maternal smoking during pregnancy (no smoking, 0–10 cigarettes per day, >10 cigarettes per day), maternal age at birth (years), maternal pre-pregnancy BMI, maternal educational level (primary school or more), and parity (order among siblings). We used the formula (10^(β)^−1) ×100 to present percentage differences in the dependent variable per unit increase of the independent variable (Figure 2).

**Table 1:** Characteristics of the study population given as medians (5–95% range) and percentages.

	Male (N = 1030)		Female (N = 1090)	
Early life factors	N		N	
Maternal BMI (kg/m^2^)	958	22.4(18.9–28.7)	1027	22.3(18.8–28.6)
Maternal smoking 1–10 cig/day (%)	1010	12.6%	1069	10.7%
Maternal smoking >10 cig/day (%)	1010	2.3%	1069	1.5%
Maternal cohabiting (%)	1030	97.7%	1088	96.8%
Maternal education longer than primary school	1014	39.0%	1076	37.9%
Maternal age (years)	1029	26.1(19.1–38.2)	1088	26.1(19.1–38.3)
Offspring first born (%)	1029	43.0%	1088	40.3%
Birth weight (kg)	1030	3.6(2.8–4.4)	1090	3.5(2.7–4.2)
Weight at 5 months (kg)	1030	7.7(6.6–9.1)	1090	7.2(6.0–8.6)
Weight at 1 year (kg)	1030	10.5(8.9–12.4)	1090	9.9(8.3–11.9)
BMI at 2 years (kg/m^2^)	1030	16.8(14.9–19.0)	1090	16.6(14.6–18.9)
BMI at 3 years (kg/m^2^)	1030	16.1(14.3–18.3)	1090	16.0(14.1–18.2)
BMI at 4 years (kg/m^2^)	1030	15.7(14.1–17.8)	1090	15.6(13.8–17.9)
BMI at 5 years (kg/m^2^)	1030	15.5(13.7–17.9)	1090	15.3(13.5–18.1)
				
**Outcome at 31 yr**				
BMI (kg/m^2^)	1030	24.8(20.1–31.4)	1090	23.0(18.8–33.8)
BMI >25 (%)	1030	46.8%	1090	30.1%
BMI >30 (%)	1030	7.8%	1090	9.1%
Metabolic syndrome (%)	1019	13.2%	1034	9.7%
Waist (cm)	1019	88(75–106)	1034	76(65–102)
Waist, male >94 cm, female >80 cm (%)	1019	27.0%	1034	36.2%
Waist, male >102 cm, female >88 cm (%)	1019	9.5%	1034	17.9%
Triglyceride (mmol/L)	1025	1.13(0.56–2.84)	1088	0.91(0.49–2.25)
Triglyceride>1.7 (%)	1025	22.2%	1088	11.2%
HDL (mmol/L)	1026	1.38(0.96–1.96)	1088	1.67(1.14–2.35)
HDL, male <1.03, female <1.29 (%)	1026	8.1%	1088	14.7%
Systolic blood pressure (mmHg)	1027	129(110–153)	1086	118(102–142)
Systolic blood pressure >130 (%)	1027	46.1%	1086	19.0%
Diastolic blood pressure (mmHg)	1027	80(63–100)	1084	75(58–93)
Diastolic blood pressure >85 (%)	1027	32.2%	1084	16.3%
Taking anti hypertensives	1030	1.8%	1090	1.8%
Fasting glucose (mmol/L)	1020	5.1(4.5–5.9)	1083	4.9(4.2–5.6)
Fasting glucose >5.6 (%)	1020	17.5%	1083	7.0%

BMI: Body mass index.

To categorize childhood body size, we subdivided children into 6 groups according to weight or BMI percentiles at the specific ages (<5,5–50, 50–75, 75–90, 90–95 and >95) in the study population and compared high percentile groups with children between the 5^th^ and the 50^th^ percentile using a generalized linear regression model with log link. We tested their risk of overweight (BMI above 25 kg/m^2^), obesity (BMI above 30 kg/m^2^), large waist circumference according to European cut-offs (>94 cm in males and >80 cm in women), large waist circumference according to North American cut-offs (>102 cm in males and >88 cm in women), and metabolic syndrome according to the International Diabetes Federation (with European cut-offs for waist circumference) [19]. These analyses were also studied separately in males and females, but the data were pooled due to lack of evidence of interaction. The estimates presented are adjusted for gender.

We calculated predictive values of having a BMI at the age of 5 years among the top 5 per cent for the five different outcomes. The predictive values are presented as the sensitivity, specificity, positive predictive value (PPV) and negative predictive value (NPV).

Differences in adult BMI between the group with and without sufficient growth measures and differences in childhood weight or BMI between individuals with and without metabolic syndrome were tested using Wilcoxon rank-sum test. A 5% significance level was used. Differences in the proportion of adults with metabolic syndrome between the groups with and without sufficient growth measures were tested using the chi-square test. A 5% significance level was used. Stata version 12 SE was used for all analyses.

## Results

Characteristics of the cohort by gender are displayed in Table 1. At the age of 31 years, more men than women were overweight (including obesity) (46.8% versus 30.1%), whereas the obesity prevalence was fairly similar and seen in 7.8% of men and 9.1% of women. Metabolic syndrome was observed in 13.2% of men and 9.7% of women, and when we studied the specific components of the syndrome, clear gender differences were found; the percentage of men exceeding the threshold in blood pressure, triglycerides and fasting glucose was twice the percentage of women, whereas the opposite was the case for HDL.

We studied the relationship between preschool weight or BMI and adult BMI, waist circumference, blood pressure, triglyceride, fasting glucose, and HDL. For adult BMI and waist circumference, a linear positive relation with preschool weight and BMI was found from birth and onwards. No evidence of a threshold was found. For HDL, an inverse relation was found from the age of 3 years and onwards (Figure 2). The relationship between preschool weight or BMI and triglyceride, fasting glucose, systolic and diastolic blood pressure showed a tendency of increasing preschool BMI being associated with increase in these metabolic alterations with increasing age, but the associations were very weak. Adjustments for cofactors did not change any of the results significantly. We also studied the association between a high BMI in childhood and generally used definitions of adiposity (overweight, obesity, and waist circumference) and metabolic syndrome (Table 2). Children above the 95^th^ percentile in preschool weight and BMI were at higher risk of adult obesity from the age of 1 year, and children above the 95^th^ percentile at the age of 5 years had a relative risk of obesity of 6.2(95% CI:4.2–9.3). Their risk of large waist circumference was increased from the age of 5 months, and at the age of 5 years, these children had a relative risk of a large waist circumference of 2.4(95% CI:2.0–2.9) according to the European threshold. Their risk of adult metabolic syndrome was increased from the age of 3 years, and at the age of 5 years, these children had a relative risk of metabolic syndrome of 2.5(95% CI:1.7–3.8). As expected, the associations strengthen with increasing childhood age. Childhood weight or BMI was higher among individuals with the metabolic syndrome from the age of 2 years compared to individuals without the metabolic syndrome. Predictive values at the age of 5 years are shown in table 3 for children above the 95^th^ percentile. PPVs show that two thirds became overweight as adults, one in three became obese, and one in four developed the metabolic syndrome.

**Table 2:** Relative risk of generally used adiposity measures and metabolic syndrome at 31 years linked to percentile division of weight at 5 months and 1 year and BMI from 2–5 years.

	Overweight		Obese		Large waist (eur.)		Large waist (USA)		Metabolic syndrome	
	n(%)	RR	n(%)	RR	n(%)	RR	n(%)	RR	n(%)	RR
Weight 5 months (kg)										
<5 perc	31(29)	1.0(0.7–1.3)	10(9)	1.2(0.6–2.2)	33(33)	1.1(0.8–1.5)	14(14)	1.0(0.6–1.7)	13(13)	1.3(0.8–2.2)
≥5–<50	306(32)	1	72(8)	1	262(29)	1	108(12)	1	101(11)	1
≥50–<75	230(43)	1.3(1.1–1.4)	49(9)	1.3(0.9–1.8)	182(35)	1.3(1.1–1.5)	79(15)	1.5(1.1–1.9)	63(12)	1.0(0.8–1.4)
≥75–<90	134(42)	1.2(1.0–1.4)	29(9)	1.3(0.8–2.0)	92(30)	1.2(1.0–1.4)	47(15)	1.6(1.2–2.3)	36(12)	1.0(0.7–1.4)
≥90–<95	61(58)	1.5(1.3–1.9)	13(12)	1.8(1.0–3.1)	44(42)	1.7(1.3–2.1)	20(19)	2.2(1.4–3.4)	12(11)	0.9(0.5–1.6)
≥95	48(45)	1.2(1.0–1.6)	6(6)	0.8(0.4–1.8)	36(34)	1.3(1.0–1.8)	14(13)	1.5(0.9–2.4)	14(13)	1.1(0.6–1.8)
Weight 1 year (kg)										
<5 perc	39(37)	1.3(1.0–1.6)	12(11)	1.5(0.8–2.6)	37(37)	1.2(0.9–1.6)	19(19)	1.5(1.0–2.4)	12(12)	1.2(0.7–2.1)
≥5–<50	307(32)	1	71(7)	1	263(29)	1	99(11)	1	97(11)	1
≥50–<75	213(40)	1.2(1.0–1.3)	39(7)	1.0(0.7–1.5)	157(30)	1.1(0.9–1.3)	71(14)	1.4(1.1–1.9)	63(12)	1.1(0.8–1.5)
≥75–<90	136(43)	1.2(1.0–1.4)	33(10)	1.5(1.0–2.2)	105(34)	1.3(1.1–1.6)	53(17)	1.9(1.4–2.6)	36(12)	1.0(0.7–1.5)
≥90–<95	54(51)	1.4(1.2–1.7)	10(9)	1.3(0.8–2.5)	39(38)	1.4(1.1–1.9)	18(17)	2.0(1.3–3.2)	13(13)	1.1(0.6–1.9)
≥95	61(58)	1.6(1.3–1.9)	14(13)	1.9(1.1–3.3)	48(46)	1.7(1.4–2.2)	22(21)	2.4(1.6–3.6)	18(17)	1.5(0.9–2.4)
BMI at 2 years (kg/m^2^)										
<5 perc	24(23)	0.7(0.5–1.1)	4(4)	0.5(0.2–1.4)	26(26)	0.9(0.6–1.2)	8(8)	0.6(0.3–1.3)	13(13)	1.3(0.8–2.3)
≥5–<50	305(32)	1	66(7)	1	263(29)	1	106(12)	1	92(10)	1
≥50–<75	211(40)	1.2(1.1–1.4)	42(8)	1.2(0.8–1.7)	159(31)	1.1(0.9–1.3)	69(13)	1.2(0.9–1.6)	65(13)	1.2(0.9–1.7)
≥75–<90	155(49)	1.5(1.3–1.7)	39(12)	1.8(1.2–2.6)	113(37)	1.3(1.1–1.5)	59(19)	1.7(1.3–2.3)	43(14)	1.4(1.0–1.9)
≥90–<95	55(51)	1.6(1.3–1.9)	12(11)	1.6(0.9–2.9)	41(39)	1.4(1.1–1.8)	21(20)	1.8(1.2–2.7)	13(13)	1.2(0.7–2.1)
≥95	60(57)	1.7(1.4–2.1)	16(15)	2.2(1.3–3.7)	47(46)	1.6(1.3–2.0)	19(18)	1.7(1.1–2.6)	13(13)	1.3(0.7–2.1)
BMI at 3 years (kg/m^2^)										
<5 perc	22(21)	0.7(0.5–1.1)	0(0)	0.0(0.0–0.0)	23(23)	0.8(0.5–1.2)	5(5)	0.4(0.2–1.0)	11(11)	1.2(0.7–2.2)
≥5–<50	287(30)	1	55(6)	1	249(27)	1	98(11)	1*	84(9)	1
≥50–<75	229(43)	1.4(1.2–1.6)	53(10)	1.8(1.2–2.5)	171(33)	1.2(1.1–1.5)	73(14)	1.4(1.0–1.8)	76(15)	1.6(1.2–2.1)
≥75–<90	151(47)	1.5(1.3–1.8)	30(9)	1.7(1.1–2.5)	109(35)	1.3(1.1–1.6)	57(18)	1.8(1.3–2.4)	35(11)	1.2(0.8–1.7)
≥90–<95	55(52)	1.8(1.4–2.1)	19(18)	3.1(1.9–5.0)	42(42)	1.5(1.2–1.9)	21(21)	1.8(1.2–2.8)	15(15)	1.6(1.0–2.7)
≥95	66(62)	2.0(1.7–2.3)	22(21)	3.6(2.3–5.7)	55(53)	2.0(1.6–2.4)	28(27)	2.6(1.9–3.8)	18(17)	1.9(1.2–3.0)
BMI at 4 years (kg/m^2^)										
<5 perc	16(15)	0.5(0.3–0.8)	1(1)	0.2(0.0–1.4)	18(18)	0.6(0.4–1.0)	7(7)	0.7(0.3–1.4)	7(7)	0.7(0.3–1.4)
≥5–<50	281(29)	1*	46(5)	1	247(27)	1	87(9)	1	94(10)	1
≥50–<75	214(40)	1.3(1.2–1.5)	47(9)	1.9(1.3–2.7)	154(30)	1.1(1.0–1.3)	65(13)	1.4(1.0–1.9)	58(11)	1.1(0.8–1.5)
≥75–<90	162(51)	1.7(1.5–1.9)	35(11)	2.3(1.5–3.5)	121(39)	1.5(1.2–1.7)	62(20)	2.1(1.6–2.9)	42(13)	1.3(0.9–1.8)
≥90–<95	64(60)	2.0(1.7–2.4)	17(16)	3.3(2.0–5.6)	47(46)	1.7(1.3–2.2)	24(23)	2.5(2.7–3.7)	14(14)	1.3(0.8–2.2)
≥95	73(69)	2.2(1.9–2.6)	33(31)	6.4(4.3–9.5)	62(61)	2.2(1.8–2.7)	37(37)	3.7(2.7–5.1)	24(24)	2.4(1.6–3.5)
BMI at 5 years (kg/m^2^)										
<5 perc	15(14)	0.5(0.3–0.8)	1(1)	0.2(0.0–1.4)	15(15)	0.6(0.3–0.9)	3(3)	0.3(0.1–0.9)	5(5)	0.5(0.2–1.2)
≥5–<50	285(30)	1*	46(5)	1	238(27)	1	87(9)	1	89(10)	1
≥50–<75	208(39)	1.3(1.1–1.5)	38(7)	1.5(1.0–2.3)	170(33)	1.3(1.1–1.5)	66(13)	1.4(1.0–1.9)	62(12)	1.2(0.9–1.7)
≥75–<90	158(50)	1.6(1.4–1.8)	41(13)	2.7(1.8–4.0)	111(35)	1.4(1.1–1.7)	60(19)	2.1(1.6–2.8)	42(13)	1.4(1.0–1.9)
≥90–<95	68(64)	2.1(1.8–2.5)	21(20)	4.1(2.5–6.5)	51(50)	1.8(1.5–2.3)	27(26)	2.6(1.8–3.9)	16(16)	1.6(1.0–2.7)
≥95	76(72)	2.3(2.0–2.6)	32(30)	6.2(4.2–9.3)	64(63)	2.4(2.0–2.9)	39(38)	3.9(2.8–5.3)	25(25)	2.5(1.7–3.8)

*Statistically significant difference between genders.

BMI – body mass index. Large waist (eur)- European cut-offs for waist circumference (male >94 cm and female >80 cm). Large waist (USA)- USA cut-offs for waist circumference (male >102 cm and female >88 cm).

**Table 3:** Predictive values of 5 year BMI in the top 5% with different outcomes.

Outcome	Sensitivity	Specificity	PPV	NPV
Adult overweight	9.4	97.7	71.7	63.6
Adult obesity	17.9	96.2	30.2	92.7
Large waist (eur)	9.9	97.3	62.8	70.0
Large waist (USA)	13.8	96.4	38.2	87.5
Metabolic syndrome	10.5	95.8	24.5	89.0

Sensitivity, specificity, positive predictive value (PPV) and negative predictive value (NPV).

Large waist (eur)- European cut-offs for waist circumference (male >94 cm and female >80 cm). Large waist (USA)- USA cut-offs for waist circumference (male >102 cm and female >88 cm). PPV- positive predictive value. NPV – negative predictive value.

The relative risk of children above the 90^th^ percentile at the age of 5 years of individual components of the metabolic syndrome revealed a tendency of increased risk of all risk indicators. The risk of elevated triglycerides, systolic and diastolic blood pressure reached statistical significance (Table 4).

**Table 4:** Relative risk of individual components of the metabolic syndrome at 31 years linked to percentile division of BMI at the age of 5 years.

	Triglycerides> = 1.7 mmol/L		HDL women <1.03 mmol/L men <1.29 mmol/L		Systolic blood pressure> = 130 mmHg		Diastolic bloodpressure> = 85 mmHg		Fasting glucose> = 5.6 mmol/L	
	n(%)	RR	n(%)	RR	n(%)	RR	n(%)	RR	n(%)	RR
BMI at 5 years (kg/m^2^)										
<5 perc	12(11)	0.7(0.4–1.3)	8(7)	0.6(0.3–1.3)	32(30)	1.0(0.7–1.3)	21(20)	0.8(0.5–1.2)	15(14)	1.4(0.8–2.2)
≥5–<50	157(16)	1	107(11)	1	317(33)	1	246(26)	1*	108(11)	1
≥50–<75	82(16)	0.9(0.7–1.2)	58(11)	1.0(0.7–1.4)	203(38)	1.1(0.9–1.2)	147(28)	1.0(0.9–1.2)	65(12)	1.1(0.8–1.4)
≥75–<90	53(17)	1.0(0.7–1.3)	43(14)	1.3(0.9–1.7)	100(32)	0.9(0.8–1.1)	81(26)	0.9(0.8–1.2)	35(11)	0.9(0.6–1.3)
≥90–<95	26(25)	1.5(1.1–2.2)	11(10)	0.9(0.5–1.6)	45(42)	1.3(1.1–1.6)	30(28)	1.1(0.8–1.5)	15(14)	1.3(0.8–2.2)
≥95	20(29)	1.2(0.8–1.8)	16(15)	1.3(0.8–2.1)	41(39)	1.1(0.9–1.5)	37(35)	1.3(1.0–1.7)	16(15)	1.4(0.9–2.3)

*Statistically significant difference between genders.

BMI – body mass index.

We tested for differences in adult BMI in the population with and without sufficient growth measures. The population with sufficient growth measures had a lower median adult BMI (23.8 versus 24.2, P = 0.002). We found no difference in the proportion with metabolic syndrome between these subpopulations.

When adjusting for adult BMI (Figure S1) the associations between preschool BMI and adult waist circumference, glucose, blood pressure, HDL and triglyceride all reversed.

## Discussion

### Main Findings

In this population-based cohort study, we found weight and BMI from birth to 5 years to be linearly associated with adult BMI and waist circumference, and BMI from 3–5 years to be inversely associated with adult HDL. We found no evidence of a threshold. The associations between preschool BMI and blood pressure and glucose were very weak. Having a BMI above the 90^th^ percentile from 3–5 years was associated with adult obesity, central obesity, and metabolic syndrome. Of children above the 95^th^ percentile in 5 year BMI, we found a third to be obese at the age of 31 years and a fourth to have metabolic syndrome.

Our results strongly indicate that at least preschool BMI from 3–5 years is indeed associated with adverse metabolic alterations in adulthood.

### Comparison with other studies

In accordance with existing evidence [20]–[22], we found higher birth and infancy weight to be associated with adult BMI. However, the association between infancy weight and metabolic alterations is complex. According to the ‘Fetal Origins Hypothesis’, coronary heart disease, and the diseases related to it, originates through responses to growth restriction during fetal life and infancy [23]. Our data indicate that higher birth and infancy weight could be associated with decreased triglycerides, glucose and systolic blood pressure supporting this hypothesis (Figure 2).

Other studies have previously found BMI in preschool ages to be linked to adult waist circumference [24], [25] and BMI change from 2 to 6 years has been linked to metabolic alterations (blood pressure, HDL and triglycerides) in adulthood [14]. Fall et al. failed to detect significant linear associations between simple childhood BMI measures before the age of 5 years and individual metabolic measures in adulthood [24], but significant associations have been found at BMI rebound [15]and among older children [2], [24].

### Strengths and Limitations

This study has several strengths. Firstly, it included a large number of children with extensive information about clinically relevant risk indicators and followed prospectively from pregnancy until adulthood. Secondly, numerous height and weight measurements made it possible to fit growth curves and to estimate the size of the child at any point of time in early childhood. One limitation was that some children had an insufficient number of measurements for growth modelling, which restricted the study population size. The fact that included cases had slightly lower median BMI suggests that this study has been done on a healthier subpopulation. In support of this, individuals with only a basic education and individuals with a history of unemployment have formerly been reported to be slightly underrepresented in the cohort [26]. Consequently, we might have underestimated the associations. The simple approach of studying the preschool age groups separately that we chose to make the results directly applicable to clinical practice, can also be seen as a limitation; i.e. a more complex path method could have provided a more comprehensive view [27]. Furthermore, data on waist circumference in childhood, paternal BMI and family history of metabolic syndrome or diabetes would have been interesting to elucidate whether such simple and easy obtainable information could add to the identification of children at risk of adult metabolic alterations.

### Metabolic syndrome

At the age of 31 years, 13% of men and 10% of women met the criteria for metabolic syndrome. The cause of the syndrome remains obscure, but the ultimate importance of metabolic syndrome is that it helps identify individuals at high risk of both type 2 diabetes and cardiovascular disease [16], [19]. Suffering from metabolic syndrome, already at the age of 31, results in exposure to the strain of metabolic alterations for decades, if nothing is done. If the consequences of the metabolic syndrome are to be prevented, preventive work will most likely be more effective if the development of the metabolic syndrome can be prevented from childhood, compared to the effects of preventive work starting, when the metabolic syndrome has already developed.

### Adjustment for adult BMI

In many studies of the association between childhood overweight and metabolic alterations, adjustment for adult BMI has been done with the purpose of “isolating” the effect of childhood BMI [1], [10], but this strategy is subject to discussion. It has been argued that associations disappear or become reverse, when adjusted for adult BMI partly attributed to statistical artifacts [28]. BMI and waist circumference are highly correlated, correlation coefficient being above 0.85 in this cohort, which makes the interpretation of results of adult waist circumference after adjustment for adult BMI difficult. However, the fact that adult metabolic alterations are closely linked to adult BMI is obvious. If the association between preschool overweight and adult metabolic alterations is dependent on adult BMI, preventive initiatives are even more important in order to prevent the tracking of childhood overweight and obesity into adulthood.

### Conclusion

High preschool BMI is consistently associated with adult obesity, central obesity, and early onset metabolic syndrome. Routinely collected measures of body size in preschool ages can help to identify children in need of focused prevention due to their increased risk of adverse metabolic alterations in adulthood.

## Supporting Information

Figure S1 **Relationship between preschool body size and adult waist circumference, blood pressure, HDL, glucose and triglyceride adjusted for adult body mass index (BMI).** Differences in the dependent variable in adulthood per kg or BMI unit in childhood are shown as percentages (x axis). We used the formula (10^(β)^−1) ×100 to present percentage differences. Unadjusted associations (solid dots) and associations adjusted for birth weight, gestational week, maternal smoking during pregnancy, maternal age at birth, maternal pre-pregnancy BMI, maternal education and parity (circles) are presented with their 95% confidence interval.
(TIF)

File S1 **Description of the growth modelling.**
(DOCX)
