# Supplementary figures and images for: Preschool Weight and Body Mass Index in Relation to Central Obesity and Metabolic Syndrome in Adulthood
Source: PLoS One. 2014 Mar 3;9(3):e89986. doi: 10.1371/journal.pone.0089986 (PMC3940896; doi:10.1371/journal.pone.0089986)

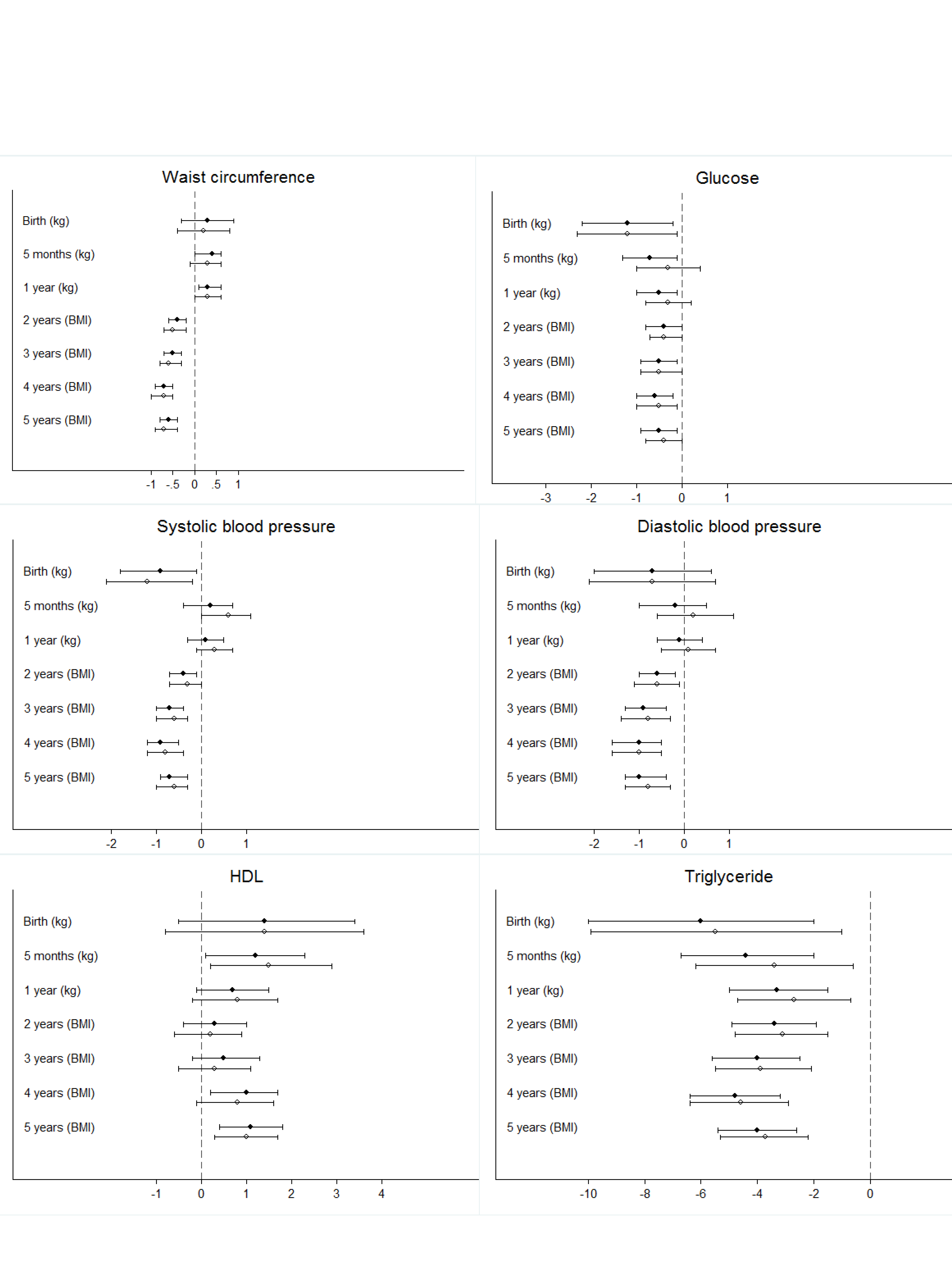

Supplement: Figure S1 — Relationship between preschool body size and adult waist circumference, blood pressure, HDL, glucose and triglyceride adjusted for adult body mass index (BMI). Differences in the dependent variable in adulthood per kg or BMI unit in childhood are shown as percentages (x axis). We used the formula (10(β)−1) ×100 to present percentage differences. Unadjusted associations (solid dots) and associations adjusted for birth weight, gestational week, maternal smoking during pregnancy, maternal age at birth, maternal pre-pregnancy BMI, maternal education and parity (circles) are presented with their 95% confidence interval. (TIF) [file pone.0089986.s001.tif]
