# Supplementary material for: Preschool Weight and Body Mass Index in Relation to Central Obesity and Metabolic Syndrome in Adulthood
Source: PLoS One. 2014 Mar 3;9(3):e89986. doi: 10.1371/journal.pone.0089986 (PMC3940896; doi:10.1371/journal.pone.0089986)
Supplement: File S1 — Description of the growth modelling. (DOCX) [file pone.0089986.s002.docx]

**File S1**

*Growth modelling*

To study the selected early weight and BMI measurements, we fitted growth curves and estimated weight and height at specific ages: 5 months and, 1-5 years. On average, 20 height and weight measurements per child in both cohorts were available from birth until adolescence. Individual growth curves for each child were fitted using the model: y = a+bt+cln(t+1)+d/(t+1) [1], where y is the growth parameter (weight or height), t is age and a, b, c, d are the parameters related to 1) baseline height/weight, 2) the linear component of growth velocity, 3) the decrease in growth velocity over time, and 4) the inflection point that allows growth velocity to peak [2].

References

1. Hauspie R, Cameron N, Molinari L. (2004) Methods in human growth research. Cambridge, UK: Cambridge University Press. xiv, 399 s., ill. p.

2. Sovio U. (2010)
Genetic variation, growth and metabolic phenotypes in the longitudinal northern finland birth cohort 1966. .
